# Organelle Interaction and Drug Discovery: Towards Correlative Nanoscopy and Molecular Dynamics Simulation

**DOI:** 10.3389/fphar.2022.935898

**Published:** 2022-06-20

**Authors:** Zhiwei Yang, Zichen Zhang, Yizhen Zhao, Qiushi Ye, Xuhua Li, Lingjie Meng, Jiangang Long, Shengli Zhang, Lei Zhang

**Affiliations:** ^1^ MOE Key Laboratory for Nonequilibrium Synthesis and Modulation of Condensed Matter, School of Physics, Xi’an Jiaotong University, Xi’an, China; ^2^ School of Life Science and Technology, Xi’an Jiaotong University, Xi’an, China; ^3^ School of Chemistry, Xi’an Jiaotong University, Xi’an, China; ^4^ Instrumental Analysis Center, Xi’an Jiaotong University, Xi’an, China

**Keywords:** organelle interaction, subcellular structure, drug discovery, nanoscopy, molecular dynamics simulation

## Abstract

The inter-organelle interactions, including the cytomembrane, endoplasmic reticulum, mitochondrion, lysosome, dictyosome, and nucleus, play the important roles in maintaining the normal function and homeostasis of cells. Organelle dysfunction can lead to a range of diseases (e.g., Alzheimer’s disease (AD), Parkinson’s disease (PD), and cancer), and provide a new perspective for drug discovery. With the development of imaging techniques and functional fluorescent probes, a variety of algorithms and strategies have been developed for the ever-improving estimation of subcellular structures, organelle interaction, and organelle-related drug discovery with accounting for the dynamic structures of organelles, such as the nanoscopy technology and molecular dynamics (MD) simulations. Accordingly, this work summarizes a series of state-of-the-art examples of the recent progress in this rapidly changing field and uncovering the drug screening based on the structures and interactions of organelles. Finally, we propose the future outlook for exciting applications of organelle-related drug discovery, with the cooperation of nanoscopy and MD simulations.

## Introduction

**FIGURE 1:**
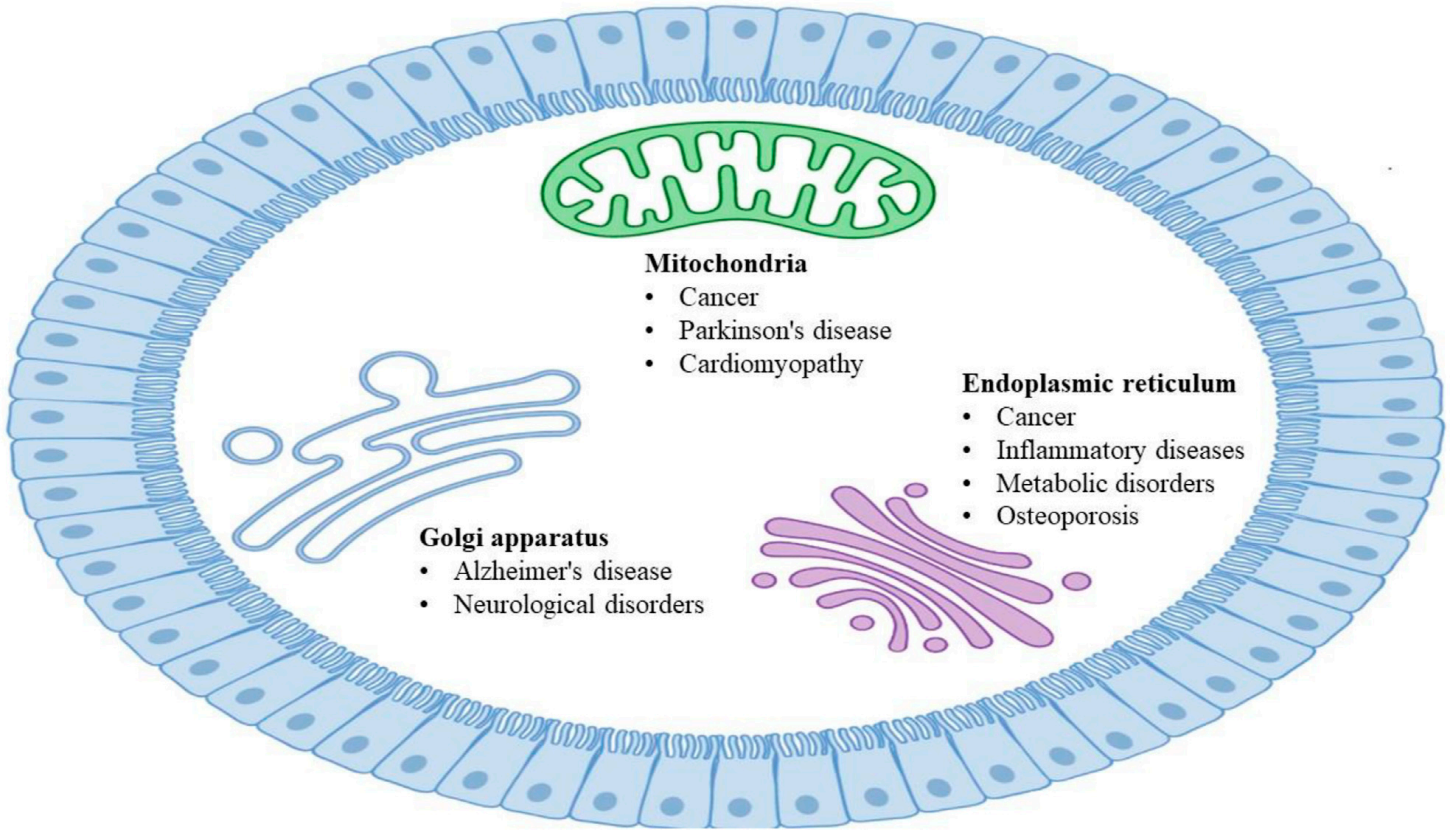
Diseases associated with the specific organelles.

Cellular organelles with specific morphology and functions are highly dynamic in maintaining the normal operation of eukaryotic cell life activities (Xu et al., 2016; Passmore et al., 2021), and they interact with each other through coordination to complete a series of important physiological functions (Valm et al., 2017). The fine division of labor, cooperation, and close contact of organelles from the interaction network to realize rapid exchanges of substance and information and carry out various biological processes under different conditions (Schwarz and Blower, 2016). Dysfunctional interactions between organelles are usually accompanied by serious diseases (Sakhrani and Padh, 2013), including Alzheimer’s disease (AD) (Santos et al., 2010; Burte et al., 2015; Wong et al., 2018), Parkinson’s disease (PD) (Hauser and Hastings, 2013; Jin et al., 2014; Pickrell and Youle, 2015; Burbulla et al., 2017), and cancer (Doria et al., 2013; Nixon, 2013; Huang et al., 2016; Mc Donald and Krainc, 2017; Plotegher and Duchen, 2017). The dysfunction of organelles in various human diseases (Figure 1) could be mechanistically resolved by studying their architectures and interactions, as well as closely monitoring the dynamic alterations (Plotegher and Duchen, 2017; Samanta et al., 2019).

Organelle bioimaging can aid our understanding of the organelle functions and the development of organelle-targeting therapy for various diseases. Conventional fluorescence microscopy (FM) opens the door to fine structural details of cellular architectures and dynamics, while the resolution is limited to approximately 200 nm because of the light diffraction (Cox and Sheppard, 2004). As the dimension of the interaction between organelles is much smaller than the light diffraction limit, such as autophagosome, mitochondria-lysosome contact, and transport vesicles, many methods and techniques surpassing the diffraction limit have been developed (Carrington et al., 1995; Huang et al., 2008; Patterson et al., 2010). Among these technologies, super-resolution microscopy (SRM) (Reiter et al., 2011) and cryo-electron microscopy (cryo-EM) (Bai et al., 2015) have established their roles in overcoming these limits and allowing the study on organelles to enter the nanoage, helping us to elucidate the dynamics structures of organelles and present the intrinsically dynamic behavior of organelle interactions.

With the status of interactions between organelles in the improvements of pathogenesis and therapeutics, related articles are emerging as an endless stream. Recent advances in organelle-targeted fluorescent probes (FPs) provide us with a more suitable selection and high resolution scale under SRM (Ning et al., 2017). Meanwhile, some review articles have deliberated on the pathways to mitochondria-lysosome interactions (Audano et al., 2018) and molecules or ions transports between mitochondria and lysosomes (Raffaello et al., 2016; Todkar et al., 2017). In this review, various strategies will be summarized to the introduction and application of nanoscopy (SRM and cryo-EM) and molecular dynamics (MD) simulations in the dynamic nature of subcellular structures, the subcellular interactions, and the organelle-related drug discovery. In addition, this review presents the future developments working in concert towards the spatial evolution and throughput necessary for nanoscopy and MD simulations to promote the organelle-related drug pipeline.

## Nanoscopy on Organelle Interactions and Drug Discovery

### Super-resolution Microscopy

**FIGURE 2:**
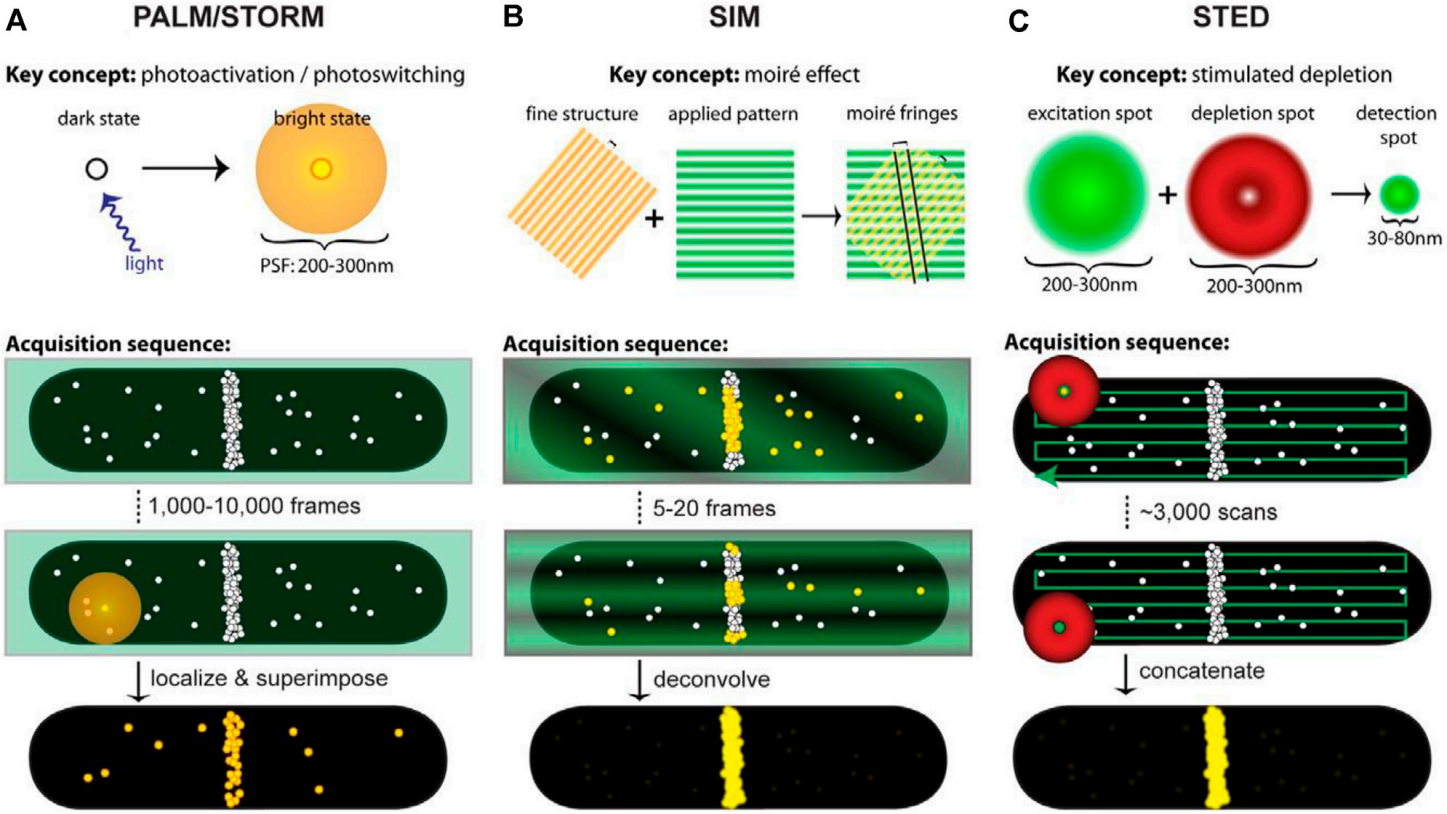
Acquisition schematics for each super-resolution technique. White circles represent molecules, green represents excitation light, red represents depletion light, and yellow highlights fluorescing molecules. In acquisition schematics, molecular positions mimic the cellular distributions of the FtsZ protein (Coltharp and Xiao, 2012). **(A)** ALM and STORM based on single-molecule localization use low levels of activation light (violet arrow) to stochastically activate and localize single molecules. An activated molecule produces a diffraction-limited spot (diffuse yellow circle) to localize the molecule’s position. Different spots are superimposed to create a superresolution image. **(B)** SIM utilizes the moiré effect. Interference between the illumination pattern (green stripes) and the sample (yellow stripes) produces moiré fringes (black lines). Although the emission from fluorescing molecules (yellow circles) is diffraction-limited, spatial information extracted from the Fourier transforms of each image with illumination patterns is combined to generate the super-resolution image. **(C)** STED projects concentric excitation (green circle) and depletion beams (red donut) onto a sample. Although the fluorophore can be excited (large green circle), the depletion beam (red donut) stimulates molecules outside the central 30–80 nm region back to the ground state before they fluoresce, generating a super-resolution PSF (small green circle). The super-resolution image is obtained by collecting beams.

Precise imaging of intracellular and subcellular structures and their dynamic processes are crucial to fundamental research in biology and medicine (Dean and Palmer, 2014; Specht et al., 2017). Super-resolution microscopy (SRM) techniques (Gustafsson et al., 2016) enable the observation of fluorescence images of subcellular organelles beyond the diffraction limit by precluding fluorescence emission when fluorophores are exposed to the excitation light, have been developed (Hell, 2007; Yang et al., 2016). More recently, SRM has been used to investigate the properties of soft matters (Woell and Flors, 2017) such as polymers (Park et al., 2015), catalysts (Peng and Long, 2011), DNA origami (Iinuma et al., 2014), and lipid-based materials (Sharonov and Hochstrasser, 2006). There are two distinct conceptual approaches to obtaining the super-resolution image. One strategy based on probes for achieving supper resolution employs stochastic activation of fluorescence to switch on individual photoactivatable molecules and then images and bleaches them at different time points, including photoactivated localization microscopy (PALM) (Betzig et al., 2006) (Figure 2A), fluorescence photoactivated localization microscopy (FPALM) (Hess et al., 2006), and stochastic optical reconstruction microscopy (STORM) (Rust et al., 2006) (Figure 2A). The other category of strategies is based on modulating the spatial pattern of fluorescence emission of molecules, including stimulated emission depletion (STED) (Zhan et al., 2017) (Figure 2C) microscopy and its generalization reversible saturable optical transitions (RESOLFT) technique (Klar et al., 2000; Gustafsson, 2005; Hell, 2007) and structured illumination microscopy (SIM) (Gustafsson, 2005; Li et al., 2015) (Figure 2B). Commercial (e.g., Volocity, Amira, and Imaris) and open-source [e.g., Fiji (Schindelin et al., 2012), ImageJ (Collins, 2007), CellProfiler (Carpenter et al., 2006), Icy (de Chaumont et al., 2011) and V3D (Peng and Long, 2011)] software packages have been developed to enable the processing and analysis of microscopy images of organelles, further reducing the difficulty of analysis. SRM can obtain the images of dynamic structures during the processes of organelle interactions, and multicolor makes it more accurate in responding to subcellular effects with the conventional fluorescent group and simple operating device. But the resolution is largely affected by the selected fluorescent probe. Besides, the introduction to fluorophore will destroy cell activity and affect the physiological environment. However, current ultra-resolution imaging methods based on light and probe can visualize the structure and dynamic processes of cells at the subcellular organelle level, which provides great possibilities for studying the pathogenesis and therapeutic of organelle-related diseases.

With the technical advancements in SRM, in practice, however, many factors can influence the achievable resolution, including the excitation and detection schemes, the properties of fluorescent probes (FPs), as well as the labeling and sampling density of FPs. SRM technologies have also enhanced the requirements of FPs, which need especially low cytotoxicity, high photostability, photobleaching resistance (Uno et al., 2014), and specific background (Han et al., 2017) to monitor organelle interplay in living cells. In recent studies, FPs (Table 1) were mainly divided into organic small-molecule probes and organelle-targeting phosphor transition metal complex probes (Kim and Cho, 2015; Wu et al., 2017). Commercial dyes (Table 2) are representative organic small-molecule probes for studying the interactions between organelles, especially in STED-based imaging. Because diluted dye solutions are used in the imaging process, the photostability of these probes leaves much to be desired. In addition, some commercial dyes alter the permeability of organelles and the inhibition of complexes at very low concentrations (Zielonka et al., 2017). Therefore, the development of new FPs is important to reveal the dynamic process of organelles for the special characters. The concept of “aggregation-induced emission” (AIE) was proposed by Tang et al. (2016) (Luo et al., 2001). Since then a series of AIE luminogens (AIEgens) can emit bright fluorescence in the aggregation state and do nothing in solution state (Zhang et al., 2019; Ni et al., 2019). Organic fluorophores for STED nanoscopy usually suffer from quenched emission in the aggregated state and inferior photostability (Dang et al., 2019). AIEgens have better photostability and photobleaching resistance than commercial dyes, so it has been considered to have great potential in STED applications (Zhang et al., 2018). TPE-Ade, a Golgi-targeting probe, was first used Ade acts as an active site of many small molecules in the Golgi apparatus and TPE with AIE characteristics, and the fluorescence intensity was enhanced by 160 times (Xing et al., 2021). Together with the good characteristics of AIE luminogens, Shen et al. synthesized whole-cell targeting nanoparticles, DTPA-BTN (Shen et al., 2021). Compared to the wide field images, the FWHM value of SIM images with DTPA-BTN was decreased by 130 and 281 nm, which increased the signal-to-noise ratios. AIEgens display good photostability and biocompatibility and can avoid fluorescence from the background. To overcome the limitations of small molecules during lysosomal membrane permeability (LMP), Xu et al. (2022) used DTPA-BT-F, an organic nanocrystal with high brightness for the lysosome imaging, at STED to monitor and long-term address lysosomal movements, including lysosomal contact. Due to the larger size, DTPA-BT-F had a diffusion limit during LMP, which prolongs their retention time with lysosomes for the long-term STED images which is the first case of AIE nanoparticles prepared by nanoprecipitation for STED. Compared with organic small-molecule probes, metal complex probes have stronger photostability, photobleaching resistance, and a large Stokes shift (Fernandez-Moreira et al., 2010). Hence, it is more suitable to observe the dynamic process of organelles for a long time (Chen et al., 2018). This provides the added advantage that lifetimes of phosphorescence are much longer than those of fluorescence, which makes changes in them potentially easier to detect (Shewring et al., 2017). Shen et al. (2018) designed and synthesized a mitochondrial-target probe LC, a Zn(II) complex based on a thiophene unit, binding with mtDNA in living cells. Due to the LC probe, the STED images recorded mtDNA distribution within mitochondrial cristae and inner matrix in living at unprecedented resolution (Shen et al., 2018). Tang et al. (2016) reported a mitochondria-targeting Zn (Ⅱ) complex dye, Znsalen J-S-Alk, whose fluorescence intensity decayed to 10% of its maximum after 360 s of continuous scanning under STORM (Tang et al., 2016).

**TABLE 1:** Properties of different fluorescent probes.

Probe	Properties
**LTR** (Zhitomirsky et al., 2018)	high quantum yield, cheap, and convenient but easily washed out, low photostability, and cytotoxicity
**MTG** (Chen et al., 2014)	
**ERTG** (Phaniraj et al., 2016)	
**TPE-Ade** (Xing et al., 2021)	used Ade acts as an active site of many small molecules and the fluorescence intensity was enhanced by 160 times
**DTPA-BTN** (Shen et al., 2021)	the FWHM value was decreased by 130 and 281 nm, which increased the signal-to-noise ratios
**LC** (Shen et al., 2018)	recorded mtDNA distribution at unprecedented resolution
**DTPA-BT-F** (Xu et al., 2022)	nanoparticle and have a diffusion limit during LMP

LTR: Lysosome Tracker Red, MTG: Mitochondria Tracker Green, ERTG: Endoplasmic Reticulum Tracker Green, TPE-Ade: Tetraphenylethylene- Adenosine, DTPA-BTN: 4,7-ditriphenylamine-[1,2,5]- thiadiazolo [3,4-c]pyridine, LC: a thiophene-based terpyridine Zn(II) complex, DTPA-BT-F: 4,4 '-(5,6-difluorobenzo[c][1,2,5]thiadiazole-4,7-diyl)bis(N,N-bis(4-methoxyphenyl)aniline).

**TABLE 2:** Common commercial probes.

Organelle	Probes
**Mitochondrion**	Mito-Tracker Green FM(Pendergrass et al., 2004)
	Mito-Tracker Red FM(Buravkov et al., 2014)
**Nuclear**	DAPI (Castanheira et al., 2009)
	Hoechst 33342 (Zhang et al., 1999)
**ER**	ER-Tracker Green (Zhang et al., 2019b)
	ER-Tracker Red (Wu et al., 2018)
	ER-Tracker Blue-White DPX (Chini et al., 2018)
**Lysosome**	Lyso-Tracker Green (Sintes and del Giorgio, 2010)
	Lyso-Tracker Red (Freundt et al., 2007)
**Golgi apparatus**	Golgi-Tracker Red (Li et al., 2017)

ER:Endoplasmic Reticulum.

The search for new therapies is a tedious process with long cycles and high risks (Bialer and White, 2010). In the last 20 years, technological advances in genomics (Xu et al., 2018), proteomics (Han et al., 2008), and metabolomics (Wishart, 2016) have greatly increased the number of potential therapeutic targets for a wide variety of important clinical diseases (Zhu et al., 2012; Li et al., 2018). However, there still exists a gap in quickly and effectively identifying the target compound with the best efficacy from a large number of candidate compounds with high specificity and sensitivity, which is also a difficulty in current scientific research. Organelles are highly dynamic and equipped to constantly and rapidly change their motility, positioning, morphology, and identity for different functions (Passmore et al., 2021). Highly dynamic organelle interactions at the subcellular level regulate intracellular equilibrium and homeostasis and have been considered as the important targets for drug discovery. SRM can realize the observation of organelle interactions in living cells, find possible targets for treating diseases, and then observe the influence of drugs on the target. For example, there are thousands of proteins attached to the mitochondria. However, most proteins are encoded by nuclear genes except 13 proteins controlled by mtDNA (Beattie et al., 1966). These proteins are synthesized in the cytosol and imported into mitochondria by highly conserved translocation machinery (Harbauer et al., 2014). Through the analysis of mitochondrial protein composition, over fifty proteins were found to be shared with the endoplasmic reticulum. Cellular proteins include apoptosis inducing factor (AIF) (Chiang et al., 2012), acyl-CoA: diacylglycerol acyl-transferase 2 (DGAT2) (Stone et al., 2009), and retinol dehydrogenase 10 (Rdh10) (Jiang and Napoli, 2013) trafficking from the ER to the mitochondria directly. In addition, pathogen-encoded proteins such as human cytomegalovirus (CMV) (Bozidis et al., 2008) encode viral mitochondrial-localized inhibitor of apoptosis (vMIA), hepatitis c virus (HCV) encodes the N3/4A protease, and human immunodeficiency virus 1 (HIV-1) encodes viral protein R (Vpr), which also traffics from the ER to mitochondria (Huang et al., 2012). The contacts of ER and outer mitochondrial membrane (OMM) may facilitate the transportation of proteins between the ER and mitochondria (Kornmann et al., 2009). Mitochondrial localization inhibitor of human cytomegalovirus (HCMV) vMIA protein, which is transmitted to the mitochondrial associated membrane (MAM) and ER is in contact with OMM. For visualizing vMIA association with MAM, a series of images under STED showed vMIA is distributed in clusters (Bhuvanendran et al., 2014). The distribution established the ability of super-resolution imaging to provide valuable insight into viral protein localization, particularly in the sub-mitochondrial compartments, and into drug discovery and medical treatment of Cytomegalovirus. In contrast to direct transport from the cytosol to the OMM and vMIA traffics sequentially from the ER to mitochondria through mitochondria-associated membrane contacts between the two organelles rather than direct transport from the cytosol to the outer mitochondrial membrane. To investigate the role of host proteins in vMIA trafficking from the ER to mitochondria, Salka et al. (2017) designed a series of experiments, and the results revealed that the Mitofusin (Mfn1/2)- and phosphofurin acidic cluster sorting protein 2 (PACS-2)-mediated ER-mitochondria tethering is not required for the ER-mitochondria trafficking, proven by a fluorescence lifetime comparison of PACS-2- and Mfn1/2-knockdown human primary fibroblasts and mouse embryos *via* STED method.

### Cryo-Electron Microscopy

**FIGURE 3:**
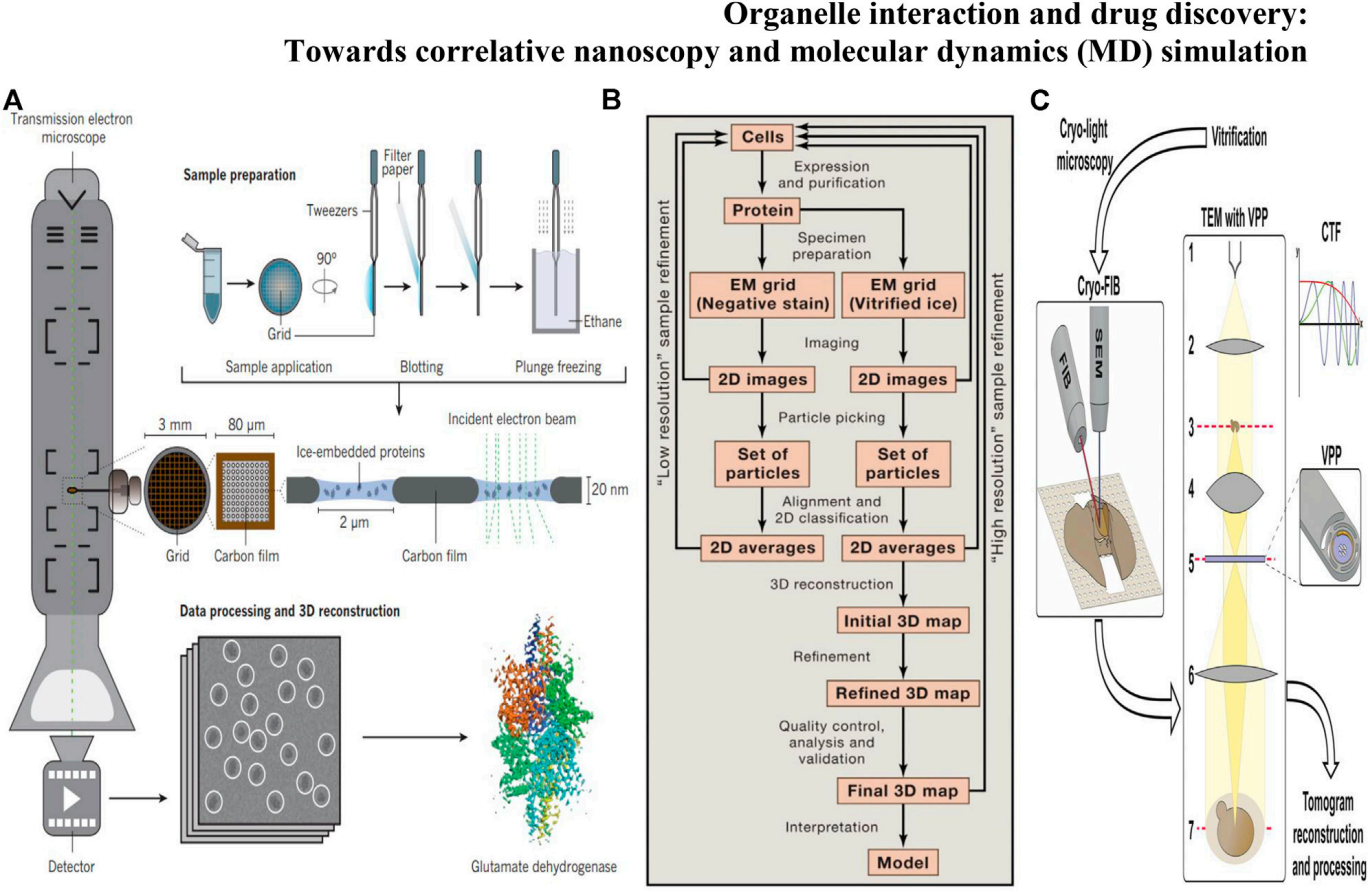
**(A)** Protein structure determination through cryo-EM involves several stages: sample grid preparation, data collection, and data processing followed by 3D reconstruction (Fernandez-Leiro and Scheres, 2016); **(B)** Steps Involved in Structure Determination by Single Particle Cryo-EM(Cheng et al., 2015); **(C)** Workflow of cellular cryo-ET by cryo-FIB milling and VPP imaging (Wagner et al., 2017).

Although SRM can achieve the imaging of subcellular structures, it is difficult to detect dense material structures by using FPs, which prohibits the understanding of organelle interactions at the nanoscale level. Cryo-electron microscopy (cryo-EM) (Glaeser, 2018) (Figure 3A) can observe hyperfine structures of organelles at near atomic resolution under the conditions closest to the physiological environment, without the need for probes to label the samples. Due to the imaging and processing processes of cryo-EM strategy, the time series of dynamic structures are difficult to extract. However, cryo-EM remains firmly established as a central tool in the arsenal of structural biology, enabling the generation of numerous near-atomic resolution structures with the highest resolution of 1.22 Å (the β3 GABA_A_ receptor) (Nakane et al., 2020). The establishment of cryo-EM benefits from the development of cryofixation through rapid cooling, which compels aqueous samples into a vitreous state, the development of efficient data processing [i.e., RELION (Zivanov et al., 2020)] and detector technology [direct electron detector, DDD (Barak et al., 2020)]. The two most prevalent approaches of cryo-EM are 1) to determine the three-dimensional (3D) structures of biological specimens: single particle analysis (SPA) (Figure 3B) (Nakane et al., 2020) and 2) cryo-electron tomography (cryo-ET) (Figure 3C) (Ng and Gan, 2020). In cryo-ET, the sample itself is imaged in 3D, and a series of 2D EM images are photographed by the sequentially tilted specimen (Wagner et al., 2017). The cryo-ET reconstruction method uses large micrographs for reconstruction, believing that large images can provide more signals and help to find the center of images at various tilting angles, but this kind of large image limits the resolution. Subtomogram averaging (STA) (Kucukelbir et al., 2014) is a recent successful development in tomography which collects data by tilt stage before tomographic reconstruction. Due to each molecule frozen in a completely random direction, there is no need to rotate the specimen stage for the projection in different directions and the projection range of SPA also exceeds the inclination range of specimen stage. The tilt angle limitation can be regarded as missing part of the sample information (Murata and Wolf, 2018). However, when the background noise is high, such as in the complex cellular environment, it is difficult to find and distinguish individual particles, only the conventional method (e.g., single particle analysis) can be adopted. In addition, the third dimension of STA is another advantage over the 2D image of SPA, because the 2D projection does not contain the absolute handedness of the structure, which increases the possibility of incorrect 3D reconstructions. STA can solve the brand-new structure with unknown symmetry and get the positive deterministic structure. However, the wrong symmetry information will cause the deviation of SPA and get the inaccurate 3D classification results.

Ren and Zhang invented the IPET method and FETR algorithm to perform the three-dimensional reconstruction. *Via* IPET and FETR, the adverse effect caused by the inclination error can be effectively limited, and the macromolecular center can be found more accurately, thus greatly improving the resolution of reconstruction results (Zhang et al., 2013). Interaction or crosstalk between organelles occurs in the blink of an eye at the nanoscale level. The dynamics of microenvironmental processes can be visualized by SRM with fluorescence probe labeling organelles however at the cost of wasting the structural and morphological information around the probes (Song and Murata, 2018). Whereas the dynamic processes of organelles are almost impossible to obtain due to the mechanism of cryo-EM forcing us to get a static picture (Ohta et al., 2021). Combining with fluorescence light microscopy and cryo-EM, correlative light and electron microscopy (CLEM) (Murata and Wolf, 2018; Ohta et al., 2021) can break through such technical limitations to meet the needs of life sciences and pharmacotherapeutics. Fluorescence microscopy captures the dynamics of the cellular process, then cell samples are fixed at a specific time. Subsequently, cryo-EM provides the surrounding ultrastructure at fluorescence localization. Focused ion beam SEM (FIB-SEM) can further improve the resolution of electron microscopy imaging of intracellular structures. Fermie was the first to use CLEM to link the dynamic characteristics and interactions of organelles to the hyperfine integrity of the structure of the labeled region. Combining CLEM and FIB-SEM, live images of the endo-lysosomal system were written down realizing the real-time tracking of late endosome-lysosome interactions (Fermie et al., 2018). In addition, Cryo-FIB provides a reliable technical option for understanding how subcellular organelles work together. Guo et al. (2022) visualized organelles of microalgae in unprecedented detail under cryo-FIB. The organelle volume of nuclear radiation mutant cells was significantly larger than that of wild-type cells.

**FIGURE 4:**
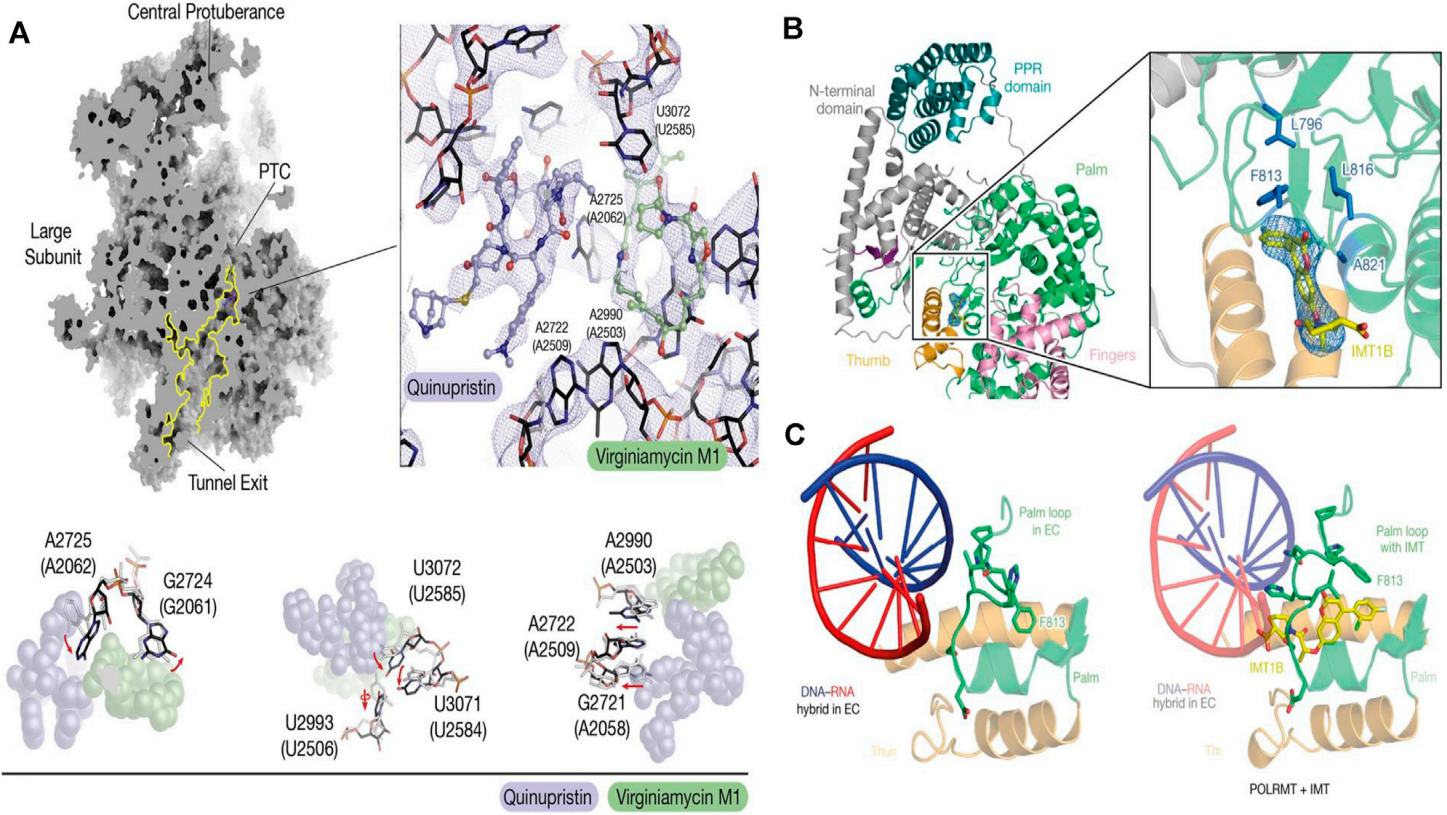
**(A)** Cryo-EM of the mitoribosome from Q/D-treated cells (Sighel et al., 2021); **(B)** Cartoon representation of the cryo-EM structure of POLRMT bound to IMT1B. The cryo-EM density for IMT1B is shown as blue mesh (Bonekamp et al., 2020); **(C)** Structure of the palm loop in the mitochondrial transcription elongation complex (EC) (PDB code: 5OLA) and the palm loop in the presence of IMT(Bonekamp et al., 2020).

The ER forms a continuous network of tubules and cisternae that extends throughout all cell compartments, including neuronal dendrites and axons (Griffing et al., 2017). There are two pathways in organelle communication: 1) vesicle transport between organelles and 2) membrane contacts without leading to the bulk transfer of organelle luminal content (Winters et al., 2020). Interorganellar communication at membrane contact sites (MCSs) plays a major role in lipid metabolism, Ca^2+^ homeostasis, and other fundamental cellular processes (Parnis et al., 2013). The ER forms MCSs with virtually all other organelles, such as the Golgi apparatus, mitochondria, lysosomes, or endosomes, as well as the plasma membrane (PM). The DHPR–RyR couplon is an excellent example of the importance of supramolecular architecture for MCS function. Visualization of components of the junction smaller than RyRs or DHPRs has been hindered by technical limitations, but pioneering cryo-ET work approached this issue in fully hydrated, unstained isolated triad junctions (Renken et al., 2009). These studies measured an average separation between sarcoplasmic reticulum and T-tubule membranes of 15.5 nm and hinted at a periodic arrangement of the calsequestrin layer, which is separated from the RyRs by a 5 nm gap bridged by fine filaments that could correspond to proteins such as triadin or junction. There are three protein coats, COPI (Zachaus et al., 2017), COPII (Hurley and Young, 2017), and clathrin (Kaksonen and Roux, 2018) mediating the formation and trafficking of vesicles in transport. The COPI coat mediates intra-Golgi and retrograde Golgi-ER trafficking and is fundamental to the polarized Golgi structure (Duden, 2003). Bykov et al. (2017) reported the *in situ* cryo-ET studies of Golgi stacks and the native structure of the COPI coat within Chlamydomonas reinhardtii cells, which provided reproducible Golgi architecture. Structural analysis of the Golgi apparatus and vesicle topology (Figure 4A) showed that vesicles change their size, membrane thickness, and cargo content as they progress from cis to trans, but the structure of the coat machinery remains constant. During apoptosis, mitochondria permeabilize the outer membranes to release apoptogenic proteins from the intermembrane space (Dingeldein et al., 2018). To further investigate mitochondrial outer membrane permeabilization (MOMP), Kuwana developed simple but faithful vesicle systems—outer membrane vesicles (OMVs) and liposomes—to visualize the pores in the membrane and dynamics by using cryo-EM *in vitro* preserving the native and hydrated membrane structure (Schafer et al., 2009; Gillies et al., 2015; Kuwana et al., 2016; Kuwana, 2019). Studies have indicated that Bax, an effector proapoptotic molecule that permeabilizes lipid membranes (Yang et al., 2006), is localized on the pore edges constituting part of the pore walls, and the pore exclusively formed by Bax oligomers will enlarge while more Bax molecules join.

Drug discovery and targeted drug transport are two links in the whole process of disease treatment. Although SRM can observe the structural changes and interaction dynamics of organelles in living cells, specific binding to organelle membrane proteins or other contact sites is still required for organelle-related drug development. Cryo-EM can image specific targets on organelles at a resolution of atomic level benefiting the understanding of therapeutic targets and drugs. By determining the structural basis for the improved affinity of the peptidic agonist of an aGPCR, peptidic antagonists toward aGPCRs were developed by converting the “finger residues” to acidic residues (Xiao et al., 2022). In search of drugs to treat hypertension and Parkinson’s disease - highly selective DRD1 agonists—Xiao et al. (2021) determined near-atomic-resolution cryo-EM structures of activated DRD1 with a downstream Gs effector revealing a conserved motif for dopaminergic receptor recognition with catecholamine agonists. With cell aging or cytopathic effect, the expression of mitochondrial DNA was changed. Inhibitors of mitochondrial transcription (IMTs) impair mtDNA transcription and inhibit mtDNA expression and the oxidative phosphorylation (OXPHOS) system. The OXPHOS system plays a vital role in the persistence of therapy-resistant cancer cell growth. Through the reconstruction of human mitochondrial RNA polymerase by cryo-EM, Bonekamp et al.(2020) found the allosteric binding site near the active center cleft of POLRMT (Figures 4B,C). After the treatment of IMT, the viability of cancer cells strongly decreased, but importantly, treatment with IMT was not cytotoxic to human PBMCs or pooled primary human hepatocytes. Prolonged treatment with an IMT thus specifically affects the proliferation of cancer cells, which suggests that cancer therapy may be a potential *in vivo* application of IMTs. Glioblastoma (GBM) is the most common malignant primary brain tumor in adults. However, existing treatments, such as surgery and chemotherapy, have little effect on glioblastoma stem cells (GSCs) (Schulze et al., 2018). After high-content screening in a custom-made library of potential mitochondrial translation inhibitors, Sighel et al. (2021) identified the bacterial antibiotic quinupristin/dalfopristin (Q/D) as an effective suppressor of GSC growth that can disrupt the cell cycle, induce cell death and inhibit the replication of GSCs. Cryo-EM results revealed that Q/D binds to the large mitoribosomal subunit, inhibiting the mitochondrial protein synthesis and functionally dysregulating the OXPHOS complexes, suggesting that Q/D could potentially be repurposed for the treatment of tumors.

## Molecular Dynamics Simulations Approach for Organelle Interactions

Although the dynamic process of organelle interactions can be obtained by nanoscopy, it is still necessary to analyze the evolution behavior at the all-atom level. Molecular dynamics (MD) simulations could obtain structural and dynamical insight into organelle interactions at the all-atom level (Fiorin et al., 2013). MD simulations have been used not only to study the dynamics of short-term organelle interactions in the presence and absence of membrane proteins but also to study the formation of structures and nanodomains around the organelle proteins. During these simulations, the coarsening models (Saunders and Voth, 2013) are usually adopted to save computing resources with the cost of sacrificing spatial resolution by allowing a significant increase in the integration time step in the numerical solution of Newton’s equation of motion (Pluhackova and Bockmann, 2015).

With the increase in understanding of organelle composition and the importance of organelle interactions, modeling of organelle interactions has become more complex. An energy-based model has been designed for the molecular reaction-diffusion dynamics involved with the cytomembrane, cytoskeletons, and organelle membranes. The existence of a clustering associated with receptor-cluster rafts and the “fluid mosaic model” in the plasma membrane was confirmed based on this perspective (Azuma et al., 2006). With the development of computer hardware and algorithms, Coarse-Grained MD simulations have been widely extended beyond the cytomembrane, entering the domain of organelles and subcellular structures (Chavent et al., 2016). A model integrating multiple data from structural biology, mass spectroscopy, and biophysics realized the near atomic resolution of synaptic vesicles (Takamori et al., 2006). Based on MD simulations of bovine heart mitochondria, Arnarez et al. (2016) revealed the reason why cardiolipins glue complexes together is cardiolipin binding strength is higher than mitochondrial lipids resulting from non-additive electrostatic and van der Waals forces, suggesting that lipids have the ability to selectively mediate protein-protein interactions. The study of interactions between membraneless organelles has also benefited from MD simulations. For example, membraneless organelles exhibit classic signatures of liquids which allows to concentrate molecular reactants and organelle interactions to take place. Wei et al. (2017) found that the effective mesh size of intracellular droplets is ∼ 3–8 nm, which determines the size scale of droplet characteristics affecting molecular diffusion and permeability, and reveals how specific intrinsically disordered proteins (IDPs) phase separate to form permeable, low-density liquid. Liquid-liquid phase separation (LLPS) condensates can simulate membraneless organelles *in vitro*. The rebalancing MD simulation force fields, based on experimental data on LLPS and without limiting specific coarse-particle sizes, not only perfect the interaction between proteins, but also correct the potential energy surface, improving the reliability of modeling interactions between membraneless organelles (Benayad et al., 2021). With the support of a new MD simulation approach equipped with a subtractive assembly technique to eliminate the overlap in space, Vermaas et al. (2022) simulated one protocell model at the organelle scale level and one protocell model at the cell scale level. The MD results revealed how membrane curvature plays a role in diffusion and protein organization at the subcellular scale level.

## Outlook

Organelle interactions play the important roles in maintaining cell homeostasis and function, and the fine organelle structures have extraordinary implications for drug discovery. Super-resolution microscopy (SRM) techniques enable the observation of fluorescence images of subcellular organelles beyond the diffraction limit and arouse the discovery of mitochondrial lysosome contact (MLC), providing a new perspective on the drug screening and the treatment of diseases (Chen et al., 2018; Chen et al., 2020). However, the resolution of ultra-resolution microscopy is still lower than that of cryo-EM, and the three-dimensional reconstruction results of organelles obtained by SRM seem to be different from those of cryo-EM approaches (e.g., cryo-ET) (Diebolder et al., 2012; Broeken et al., 2015). With the development of cryo-EM, the resolution of organelle structures has reached the approximate atomic level, especially the dynamic structural changes of the organelle interactions. While, the three-dimensional reconstruction of cryo-EM captures the configuration frozen in various random states, with the less time sequence information of captured structures.

The ideal of organelle interaction research is to capture a series of atomic resolution images in active state in chronological order, construct the dynamic structures during the interaction processes, and deeply understand the details of conformational transitions and “energy motion” transformation mechanism. Due to the limitation of current nanoscopy techniques, SRM and cryo-EM analyses cannot reach the atomic resolution or look at how a particular change over time trends. The two deficiencies could be partially compensated by molecular dynamics (MD) simulation. Although the computational power of MD simulation is far from being able to replace the experiments, through careful design, especially constrained by the key dynamic spatial and temporal data provided by nanoscopy (SRM and cryo-EM), it can provide sufficient information for the construction of the initial model. Combined with Coarse-Grained MD and all-atom MD simulations, it is expected to not only connect the dynamic evolution process of organelle interaction on a large scale (the application of CG models sacrifices degrees of freedom), but also analyze the evolution behavior of key dynamic nodes at the all-atom level (reintroducing the atomic details by subsequent all-atom models), then summarize the physical characteristics and laws of the structure-activity changes of the system, and establish the correlation between the dynamic structure and function of organelles (Marrink et al., 2019).

With the continued advances in SRM, cryo-EM, and MD simulation, the combination of the three techniques and the cooperation of super-resolution time series and cryo-EM structure information will be an effective solution for the in-depth investigation of large-scale dynamic structural evolution and will provide new insight for the organelle interaction and drug discovery. It is promising to see the breakthroughs with this approach in the field of organelle-related drug discovery.
